# Ontogeny of hepatic metabolism in mule ducks highlights different gene expression profiles between carbohydrate and lipid metabolic pathways

**DOI:** 10.1186/s12864-020-07093-w

**Published:** 2020-10-27

**Authors:** William Massimino, Stéphane Davail, Aurélie Secula, Charlotte Andrieux, Marie-Dominique Bernadet, Tracy Pioche, Karine Ricaud, Karine Gontier, Mireille Morisson, Anne Collin, Stéphane Panserat, Marianne Houssier

**Affiliations:** 1 Univ Pau & Pays Adour, INRAE, E2S UPPA, UMR 1419, Nutrition, Métabolisme, Aquaculture, F-64310 Saint Pée sur Nivelle, France; 2 IHAP, Université de Toulouse, ENVT, INRAE, UMR 1225, 31076 Toulouse, France; 3 INRAE Bordeaux-Aquitaine, UEPFG (Unité Expérimentale Palmipèdes à Foie Gras), Domaine d’Artiguères 1076, route de Haut Mauco, F-40280 Benquet, France; 4 GenPhySE, Université de Toulouse, INRAE, ENVT, F-31326 Castanet Tolosan, France; 5 INRAE, Université de Tours, BOA, 37380 Nouzilly, France

**Keywords:** Liver, Embryogenesis, Transcriptome

## Abstract

**Background:**

The production of foie gras involves different metabolic pathways in the liver of overfed ducks such as lipid synthesis and carbohydrates catabolism, but the establishment of these pathways has not yet been described with precision during embryogenesis. The early environment can have short- and long-term impacts on the physiology of many animal species and can be used to influence physiological responses that is called programming. This study proposes to describe the basal hepatic metabolism at the level of mRNA in mule duck embryos in order to reveal potential interesting programming windows in the context of foie gras production. To this end, a kinetic study was designed to determine the level of expression of selected genes involved in steatosis-related liver functions throughout embryogenesis.

The livers of 20 mule duck embryos were collected every 4 days from the 12th day of embryogenesis (E12) until 4 days after hatching (D4), and gene expression analysis was performed. The expression levels of 50 mRNAs were quantified for these 7 sampling points and classified into 4 major cellular pathways.

**Results:**

Interestingly, most mRNAs involved in lipid metabolism are overexpressed after hatching (FASN, SCD1, ACOX1), whereas genes implicated in carbohydrate metabolism (HK1, GAPDH, GLUT1) and development (HGF, IGF, FGFR2) are predominantly overexpressed from E12 to E20. Finally, regarding cellular stress, gene expression appears quite stable throughout development, contrasting with strong expression after hatching (CYP2E1, HSBP1, HSP90AA1).

**Conclusion:**

For the first time we described the kinetics of hepatic ontogenesis at mRNA level in mule ducks and highlighted different expression patterns depending on the cellular pathway. These results could be particularly useful in the design of embryonic programming for the production of foie gras.

## Background

In the context of foie gras production, better knowledge of the establishment of hepatic metabolic pathways during embryogenesis could be of particular interest to modulate the individual response to force-feeding. Indeed embryogenesis is a period of development with high plasticity which can be disturbed by environmental stimuli leading to a modification of certain physiological responses in adulthood [1, 2]. Purposefully using this process, called “embryonic programming”, can improve animal performances when a specific challenge is encountered later in life. In mule ducks, we recently demonstrated for the first time that a thermal stimulus over a period covering approximately 50% of the incubation improves the production of foie gras at the age of 3 months [3]. However some negative effects have also been observed (decrease in hatchability, slight decrease in quality of the final product) showing that a better understanding of the metabolism at the embryonic stage in ducks is needed. Therefore, even if duck embryogenesis has been well described in terms of overall morphogenesis [4–6], the specific characterization of hepatic ontogenesis at the metabolic level remains to be explored.

Liver fattening involves the activation of several metabolic pathways. First, hepatocytes must absorb circulating carbohydrates from cornstarch and catabolize glucose [7] to provide substrates for lipid synthesis via the lipogenesis pathway [8, 9]. These newly formed lipids can then be exported to the general circulation and absorbed by the peripheral tissues [10], or recaptured by the liver, thus amplifying the capacity of this organ to gain fat [11].

Therefore, the aim of the present study was to analyze a wide range of genes involved in liver development, cell stress, lipid and carbohydrate metabolisms throughout embryogenesis in mule ducks to better understand the ontogeny of pathways related to liver fattening.

Since liver sampling was only possible from the 12th day of embryogenesis (E12), we analyzed hepatic gene expression at 7 sampling points every 4 days from this point up to 4 days post-hatch (D4) and revealed different patterns of expression depending on the cellular pathway.

Interestingly, carbohydrate-related genes appear to be highly expressed at the start of kinetics, while most lipid-related genes are overexpress after hatching, revealing greater sensitivity to the food transition that occurs at this stage.

## Results

### Liver development-related gene expression

**Fig. 1:**
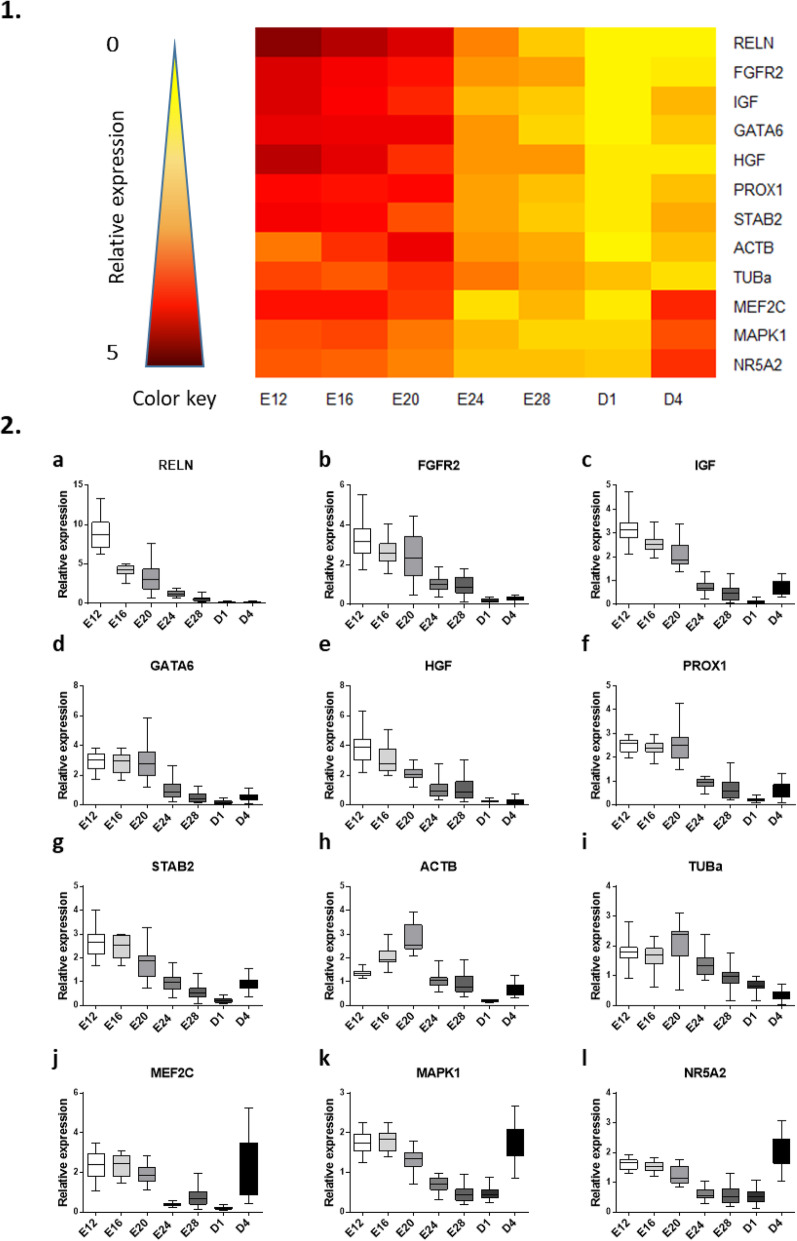
Relative hepatic expression of development-related genes from E12 to D4. 1. Heatmap illustration of liver gene expressions at different stages in mule ducks. Low gene expression is indicated in yellow, while high expression is in red, according to the color key. 2. Box-and-whisker plots representations of expression profile of RELN (**a**), FGFR2 (**b**), IGF (**c**), GATA6 (**d**), HGF (**e**), PROX1 (**f**), STAB2 (**g**), ACTB (**h**), TUBa (**j**), MEF2C (**j**), MAPK1 (**k**), NR5A2 (**l**) in the liver of mule duck during development. The boxes extend from the 25th to the 75th percentiles, and the whiskers range from the lowest value to the highest

The relative expressions of genes related to development in the liver are illustrated in Fig. 1. The heatmap representation (Fig. 1.1) clearly divided the profiles into two or even three distinct parts, the peak of expression occurring for most genes between the embryonic day 12 (E12) and the embryonic day 20 (E20) (see statistical summary in supplemental Table 1). The lowest expression level appeared mainly on the first day after hatching (D1), before a slight increase observed for most genes on the 4th day after hatching (D4). Most of these genes are involved in the processes of cell proliferation (IGF1, FGFR2), differentiation (PROX1, NR5A2) and liver development (GATA6, HGF, PROX1) (see supplemental Table 5) and their expression predominantly arose at the beginning of the kinetics.

### Carbohydrate-related gene expression

The second figure depicts the relative expression of carbohydrate-related genes. Again, the weakest expression appeared on D1, as illustrated by the heatmap (Fig. 2.1), while the mRNA level was significantly higher between E12 and E20 than at the end of kinetics for most genes (Fig. 2.2 and statistical summary in supplemental Table 2). Nonetheless, compared to development-related genes, the major peak seemed to be tighter around E20. Only the transcription factor ChREBP seemed time-shifted, with a trough at the very beginning of kinetics and a peak at E28. Genes involved in the transport of glucose (GLUT1, GLUT2) or glycolysis (GAPDH, HK1) (supplemental Table 6) were mainly expressed at the beginning of kinetics, the maximal expression occurring at E20.

**Fig. 2:**
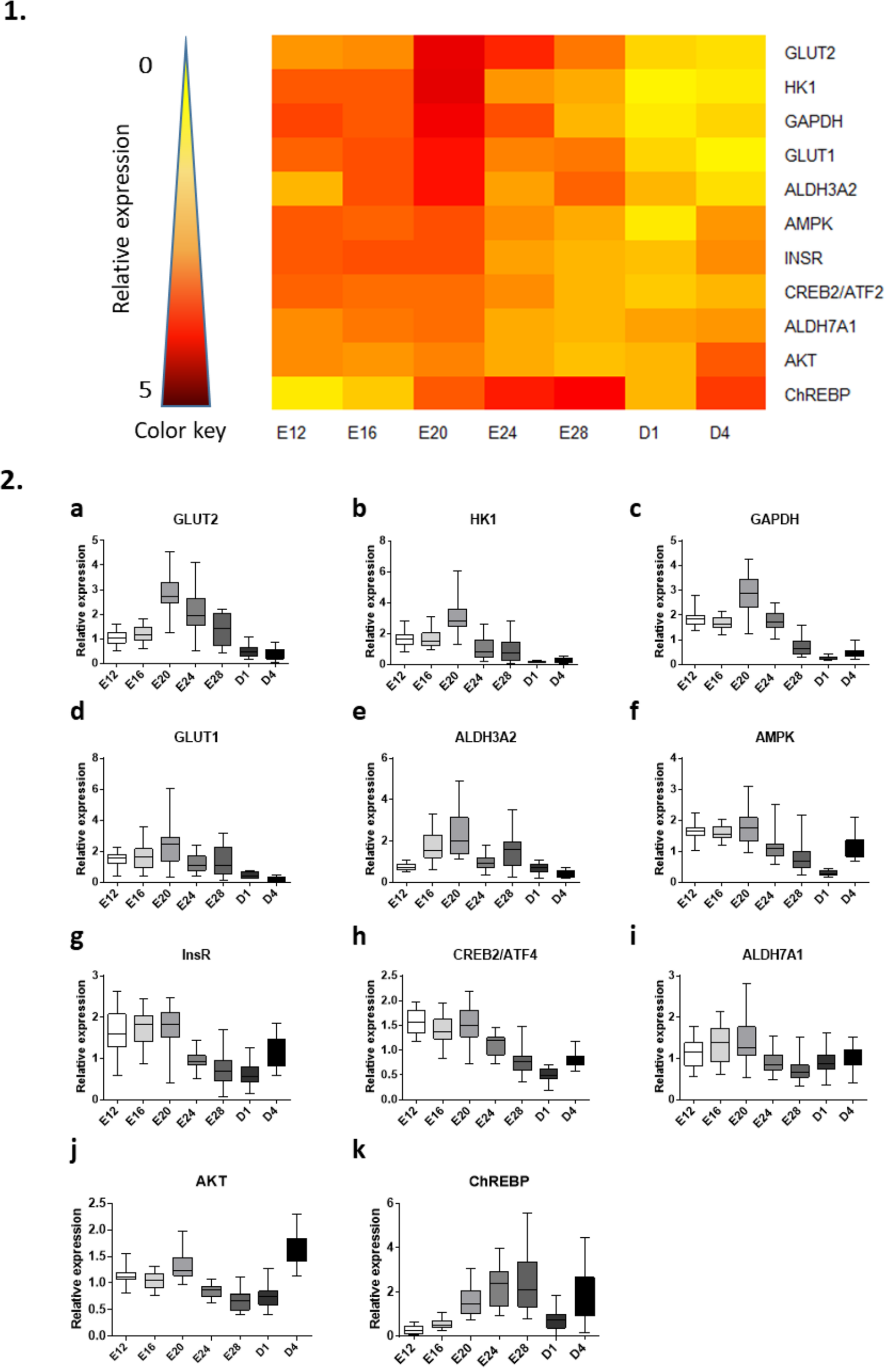
Relative hepatic expression of carbohydrate-related genes from E12 to D4. 1. Heatmap illustration of liver gene expressions at different stages in mule ducks. Low gene expression is indicated in yellow, while high expression is in red, according to the color key. 2. Box-and-whisker plots representation of expression profile of GLUT2 (**a**), HK1 (**b**), GAPDH (**c**), GLUT1 (**d**), ALDH3A2 (**e**), AMPK (**f**), INSR (**g**), CREB2/ATF2 (**h**), ALDHA7 (**i**), AKT (**j**), ChREBP (**k**) in the liver of mule duck during development. The boxes extend from the 25th to the 75th percentiles, and the whiskers range from the lowest value to the highest

### Lipid-related gene expression

The third figure reveals the expression profiles of lipid-related genes from E12 to D4. As demonstrated by the heatmap (Fig. 3.1), a clear cut appeared for all gene expressions with a sharp increase on D4 compared to the rest of the kinetics (Fig. 3.2 and supplemental Table 3), with the exception of DGAT2 and ACSS1 which displayed a profile close to that of the genes related to carbohydrate metabolism.

**Fig. 3:**
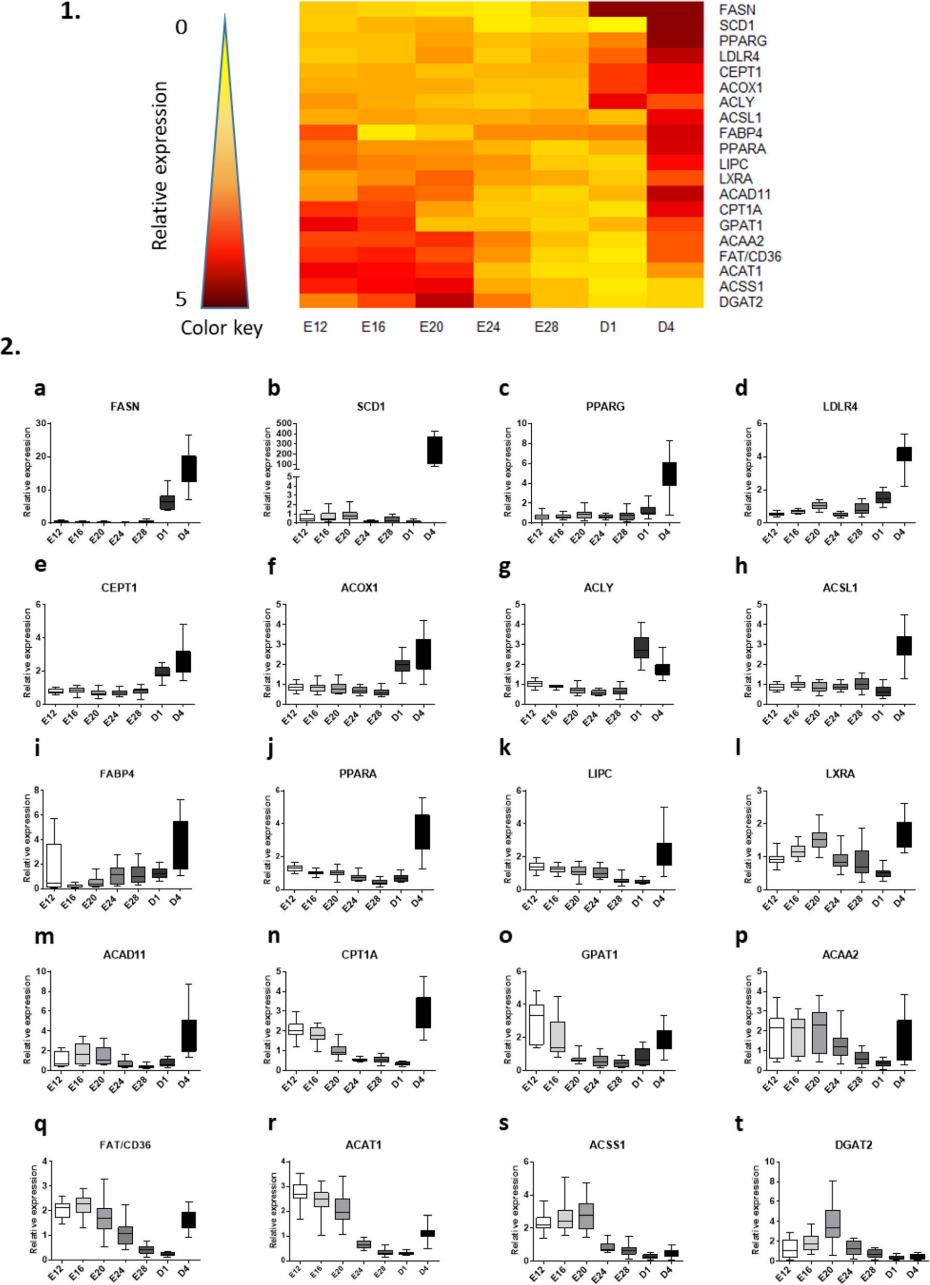
Relative hepatic expression of lipid-related genes from E12 to D4. 1. Heatmap illustration of liver gene expressions at different stages in mule ducks. Low gene expression is indicated in yellow, while high expression is in red, according to the color key. 2. Box-and-whisker plots representations of expression profile of FASN (**a**), SCD1 (**b**), PPARG (**c**), LDLR4 (**d**), CEPT1 (**e**), ACOX1 (**f**), ACLY (**g**), ACSL1 (**h**), FABP4 (**j**), PPARA (**j**), LIPC (**k**), LXRA (**l**), ACAD11 (**m**), CPT1A (**n**), GPAT1 (**o**), ACAA2 (**p**), FAT/CD36 (**q**), ACAT1 (**r**), ACSS1 (**s**), DGAT2 (**t**) in the liver of mule duck during development. The boxes extend from the 25th to the 75th percentiles, and the whiskers range from the lowest value to the highest

Most of the genes related to lipid synthesis are weakly expressed at the beginning of the kinetics, with high expression only after birth, such as FASN, SCD1, PPARG, CEPT1 or ACLY. On the other hand, several genes mainly related to lipid catabolism also show high expression at the beginning of the kinetics, such as ACAD11, CPT1A, ACAA2, or ACAT1 (Fig. 3 and supplemental Tables 3 and 7).

**Fig. 4:**
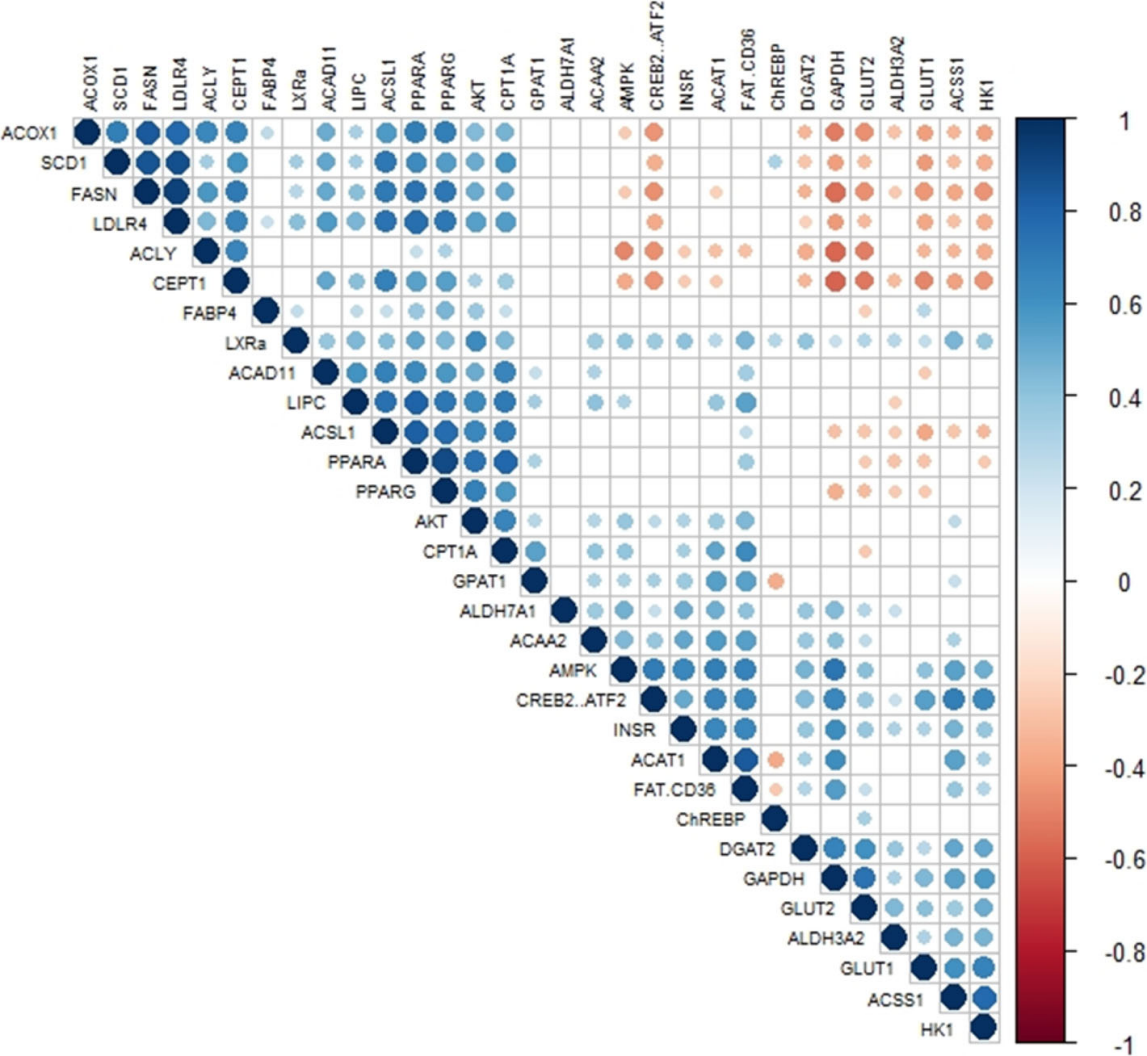
Correlation matrix of lipid and carbohydrate gene expressions. This color-coded correlation matrix illustrates the pairwise correlations between levels of gene expression throughout the kinetics (Pearson method, the presence of colored dot means *p* > 0.01). The color scale on the right indicates the strengths of the correlations (blue for positive correlation, red for negative correlation)

It is noteworthy that the correlation matrix (Fig. 4) revealed a significant negative link between a group of carbohydrate-related genes and a second group related to lipids. Indeed, ACOX1, SCD1, FASN, LDLR4, ACLY and CEPT1 appeared to be strongly negatively correlated to CREB2/ATF2, DGAT2, GAPDH, GLUT2, GLUT1 and HK1.

### Stress-related gene expression

The last figure represents the relative expression of stress-related genes. The heatmap (Fig. 5.1) underlined a peak of expression after birth for most of the genes, particularly on day 4 (Fig. 5.2 and supplemental Table 4). Several of these genes are related to heat stress (HSP90AA1 or HSBP1) or cellular detoxification (CYP2E1, GSTT1 or GSTK1) (supplemental Table 8).

**Fig. 5:**
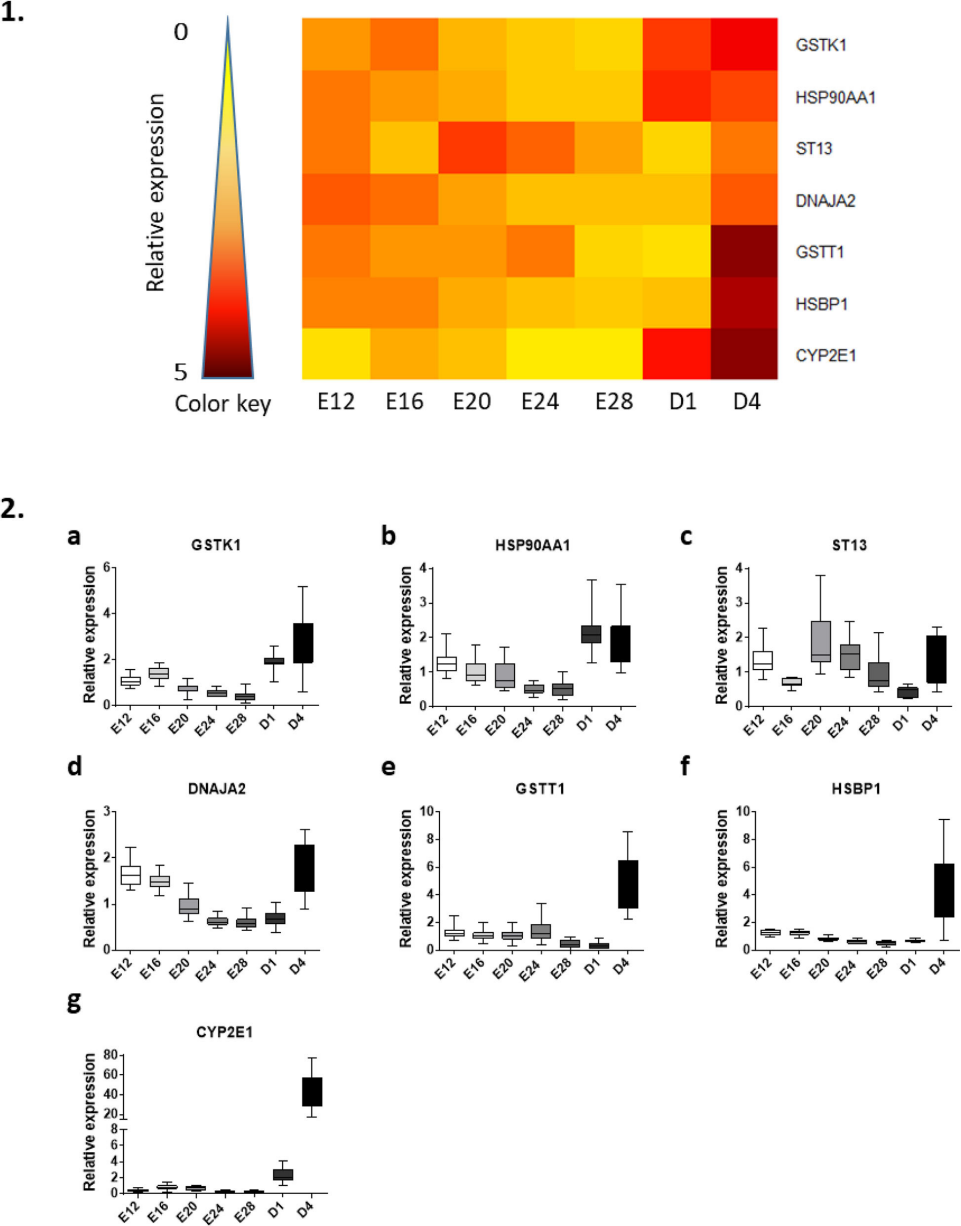
Relative hepatic expression of stress-related genes from E12 to D4. 1. Heatmap illustration of liver gene expressions at different stages in mule ducks. Low gene expression is indicated in yellow, while high expression is in red, according to the color key. 2. Box-and-whisker plots representations of expression profile of GSTK1 (**a**), HSP90AA1 (**b**), ST13 (**c**), DNAJA2 (**d**), GSTT1 (**e**), HSBP1 (**f**), CYP2E1 (**g**) in the liver of mule duck during development. The boxes extend from the 25th to the 75th percentiles, and the whiskers range from the lowest value to the highest

## Discussion

The concept of early programming is based on the high plasticity of organisms during their development, allowing them to adapt their phenotype to environmental conditions. In poultry, it has been shown that embryonic thermal programming improves the survival of animals exposed to subsequent heat stress [12], and it is particularly interesting to note that the best embryonic period to apply the stimulus corresponds to the maturation period of the hypothalamo-hypophysis-thyroid axis, which is involved in thermal regulation [13]. Remarquably, the adapted phenotype may also respond differently to new environmental challenges, such as embryonic thermal manipulation resulting in increased foie gras production in mule ducks at the age of 3 months [3]. Although the mechanisms are not yet fully understood, the timing of the application of the environmental stimulus for programming seems to be very important. In this context, it seems interesting in the field of foie gras production, to study the ontogeny of the metabolic pathways involved in liver fattening, in order to reveal potentially interesting windows of application of the thermal stimulus.

As a first step, the description of gene expression profiles in embryonic duck liver is in itself particularly informative to understand the establishment of hepatic metabolism pathways.

However, since the size of the livers did not allow sampling before E12, it is impossible to conclude on the specifically hepatic expression of developmental genes before this stage. Data on early chicken embryogenesis suggest that hepatic induction of the anterior endoderm via an interaction with the “cardiac” mesoderm [14] involves many of the pathways depicted in Fig. 1 from the very beginning of ontogeny [15, 16]. Nevertheless, although much of the cell proliferation and hepatic differentiation arise at the earliest stages of liver development [17], our results suggest that these signaling pathways (supplemental Table 5) are still strongly involved in ducks between E12 and E20, in morphogenetically distinct livers. Consequently, an environmental stimulus occurring during this period could potentially influence the proliferation and differentiation of hepatocytes, thereby causing a modification in the final number of cells in the mature organ, as previously shown for chicken muscle cells [18, 19]. Therefore, even though hyperplasia does not seem to be involved in fatty liver enlargement during overfeeding [20], it is conceivable that an increase in the number of hepatocytes at birth may enhance the fattening of the liver during force-feeding, since the ability of each cell to expand (hyperphagia) may not be affected. Moreover, recent studies [3, 21] suggest that the histological structure of the liver after overfeeding, particularly the number and size of cells, may play a role in the final quality of the product, mainly indicated by fat loss after cooking. It would therefore be very interesting to determine the precise impact of the embryonic thermal stimulus on the number of hepatic cells at birth and after overfeeding in order to accurately modulate the final yield of fatty liver through a specific programming protocol.

In oviparous animals, the nutrition of the developing embryo depends entirely on the resources from yolk and albumen. Despite the low amount of carbohydrate in the egg [22, 23], glycolysis has been described as an extremely important source of energy during the first third of chicken embryogenesis [24] and hatching [25]. The present results highlight that expression of carbohydrate-related genes is strongly committed up to E20 in mule duck embryos (Fig. 2), in particular those related to glucose transport (GLUT1 and 2) and glycolysis (GAPDH and HK1), confirming the major role of the liver in systemic glucose homeostasis throughout embryogenesis [26, 27]. Lastly, the drop in carbohydrate-related gene expression observed at D1 might reflect the decline of endogenous resources after hatching, a process involving high energy demand. Since carbohydrate metabolism is a major pathway involved in fattening the liver during overfeeding, the high expression of carbohydrate-related genes around E20 may represent an interesting period for embryonic programming by environmental stimulus. With the exception of ChREBP, the present results suggest that the programming period that may have an impact on carbohydrate metabolism could be centered around E20. Nevertheless, it is still possible that a stimulus applied up to E27 had an impact on the resulting activity of ChREBP. As a major transcription factor playing a key role in carbohydrate and lipid metabolism [28, 29], it cannot be excluded that a programming protocol applied during its peak of expression may make an important contribution to the physiological response after overfeeding. Only programming experiments with different stimulus protocols and an in-depth analysis of the impact on ChREBP mRNA and protein expressions, or activity could provide a definitive answer about its specific role and that of other carbohydrate-related genes.

With regard to the lipid metabolism, the significant overall change occurring on the 4th day after birth suggests that unlike the genes involved in carbohydrate metabolism, the expression of lipid-related genes could be strongly affected by first meals. Indeed, ducklings sampled on D1 were slaughtered before the first meal, while the ducklings sampled on D4 were all fed ad libitum since day 2. The use of yolk lipids during the development of avian embryos has been well described in a previous review [30]. These lipids are the main source of energy during the last week of embryogenesis, when the embryos exhibit an exponential growth [24, 31]. Therefore, the starting diet, mainly composed of wheat and corn, can be interpreted as a nutritional transition since the ducklings move from an energy source consisting primarily of lipids from egg yolk to an exogenous diet with high carbohydrate content [32]. This crucial transition phase is also accompanied by a major change in the metabolism of the liver that acquires the ability to synthetize its own lipids [33]. The present results, like previous studies on chickens [34, 35], illustrate this modification of hepatic lipid metabolism by highlighting the sharp increase in the expression of lipogenic genes such as SCD1 (Fig. 3.2.b) and FASN (Fig. 3.2.a) at D4 in mule ducklings. These genes are involved in the de novo lipogenesis pathway [36, 37] which reflects the ability to store carbohydrate sources as lipids [38]. In a context of nutritional change with a sudden high intake of carbohydrates, it is consistent to stimulate their storage by increasing the expression of genes involved in lipid synthesis, the liver being the predominant site of lipogenesis in birds [39, 40].

However, we observe that the pathway of lipid catabolism is also still engaged at D4, with high expression of ACOX1, ACAD11, CPT1A, ACAA2, suggesting that energy metabolism depends on the use of both carbohydrates and lipid at this stage in mule ducks. Therefore, environmental programming during this critical period could be particularly interesting to study in the context of the response to overfeeding and the production of foie gras. Finally, several genes mainly involved in lipid catabolism (PPARA, CPT1A, ACAA2, ACAT1) also showed high expression at the beginning of the kinetics, between E12 to E20. Indeed, beta-oxidation of fatty acids provides a large part of the energy demand during embryogenesis [30]. Consequently, the application of an environmental stimulus during this period could potentially program a different response to force-feeding and thus improve the phenotype.

However, the negative correlation measured between the expression of several carbohydrate and lipid-related genes during embryogenesis suggest that these two pathways, which seem to work in mirror mode during development [24, 30], could be affected differently by early-life programming. Targeting both with a thermal stimulus around E20, where most carbohydrate-related genes and some of the genes related to lipid catabolism are strongly expressed, seems to be the most appropriate choice. Nevertheless, these results also open a new programming window, around the first meals and specific to lipid-related genes, which could be interesting to explore in the context of the production of foie gras.

The overall increase in stress-related gene expressions occurred after the transfer of ducklings from the hatchery to the breeding facility, resulting in a significant temperature change from 37.3 °C to 26–28 °C. It is interesting to note that a change in the ambient temperature induced a significant increase in the hepatic expression of heat-sensitive genes involved in protein folding [41–43] (supplemental Table 8). If the thermal stimulus applied during embryogenesis induced a direct modification of their expression, it might be of interest to use them as positive markers of stimulation. Since the products of these genes are involved in the folding of different types of proteins, a change in their expression profiles could have an impact on several enzymatic activities, even those involved in metabolic processes. To answer this question, an upcoming study will focus on the immediate impact of the thermal change during embryogenesis on the expression level of these genes.

The hatching process represents a major challenge in terms of nutritional regulation, control of body temperature, but also of transition from chorioallantoic to pulmonary respiration [30, 35]. This abrupt metabolic change with the sudden onset of elevated oxygen levels may result in an increase in oxidative stress that must be controlled to maintain overall cellular homeostasis. The enhanced expression of several genes involved in cellular detoxification such as GSTK1 [44], GSTT1 [45] or CYP2E1 [46] in the liver of newborn ducklings may be a reflection of this control system. Finally, these expression patterns confirm that hatching is certainly the most brutal challenge a bird faces throughout its life and suggest that embryonic thermal stimulus could be specifically traced by some of these stress-related biological markers.

## Conclusion

These results highlight a wide range of gene expressions during liver ontogenesis in mule ducks and describe for the first time the embryonic establishment of carbohydrate and lipid metabolisms. In the context of foie gras production, the identification of these embryonic expression profiles could be of interest in order to design new programming protocols.

## Methods

### Number of animals and method of euthanasia

In accordance with Directive 2010/63/EU of the European Parliament and of the Council of 22 September 2010, all the animals were slaughtered by decapitation (as birds weighting less than 250 g), and the number of animals was reduced to the maximum by setting the power of the test at 80% and the alpha risk at 5% using a bilateral test. From previous studies of gene expression, we expected a coefficient of variability around 50%, and wanted an inter group variation of 50%. These parameters lead us to calculate *n* = 16 animals per group. Given the fertility and hatchability rates (estimated at 90 and 80% respectively), we chose to incubate 160 eggs (to assure 7 sample points of 16 individuals), and we were finally able to sample 20 individuals per group.

### Animal and sample collections

A total of 160 mule duck eggs, from mothers aged 46 weeks (genotype H85, provided by Grimaud Frères Selection Company, Roussay, France), were kept at room temperature during 3 days, prior to incubation at 37.6 °C, and 60% average relative humidity (RH) during the whole incubation period. All eggs were turned through 90° every 3 h. Temperature and hygrometry were continuously measured by a sensor (KIMO). Unfertile eggs were excluded by candling at E10, with a sliding of remaining eggs to prevent local temperature disturbances caused by the appearance of holes. At E27, all eggs were placed in the same hatcher at 37.3 °C and 80% RH. On day 2, the ducklings were transferred to a rearing room where the ambient temperature was adjusted to 26–28 °C and the starting diet (PALMA07, Maïsadour, France) was available ad libitum. Livers from 20 randomly selected animals were sampled every 4 days from 12th embryonic day (E12) to the 4th day after birth (D4).

Samples were frozen in liquid nitrogen for RNA analysis. Total RNA was isolated from frozen tissue according to the Ribozol method (VWR Life Science). Total RNA concentration was measured by spectrophotometry (optical density at 260 nm) using a Biotek EPOCH 2 microplate reader with Take3 Plate, and all the samples were normalized at 500 ng/ μl. The integrity of total RNA was analyzed by electrophoresis. An amount of 3 μg RNA was reverse-transcribed to cDNA with Iscript Reverse Transcription Supermix for RTqPCR (Bio-Rad, USA) with duplicates of samples. DNA contamination was prevented by DNase treatment. Reverse transcription reaction was done in CFX384 (Bio-Rad, USA) according to this program: 25 °C/5 min, 46 °C/20 min, 95 °C/1 min.

### qPCR EvaGreen using BioMark

The mRNA levels of 50 genes coding for proteins involved in lipid metabolism, carbohydrate metabolism, stress and development were quantified. The primer sequences (listed in supplemental Tables 5 to 8) used in the qPCR assays were created first by aligning the protein sequences of humans, mice and ducks (*Anas platyrhynchos*) on MultAlin [47] to identify the best-preserved exon. These exons were then treated on Primer3 [48, 49] to build specific primers. Validation of their efficiency ranging from 1.90 to 2 was performed using cascade dilution of a pool of cDNA, and their specificity was confirmed by sequencing the amplicon. High throughput real-time quantitative PCR was performed using the Biomark microfluidic system from Fluidigm (GeT-PlaGe platform, Castanet-Tolosan, France) in which every sample-gene combination is quantified using a 96.96 Dynamic Array™ IFCs (BMK-M-96.96, Fluidigm,). Pre-amplification of the samples, chip loading and real time quantitative PCR were performed according to manufacturer’s protocol. Real time quantitative PCR results were analyzed using the Fluidigm real-time PCR analysis software v.4.1.3.

Firstly, 6.5 ng of each cDNA were initially preamplified (10 min 95 °C activation and 14 PCR cycles (15 s 95 °C and 4 min 60 °C) with PreAmp Master Mix (100–5581, Fluidigm) and a pool containing all the primers targeting all the genes (200 nM), excluding the 16S rRNA primer sets. Preamplified sample were diluted at 1/5 after an exonuclease treatment (M02935, NEB). In order to prepare samples for loading into the integrated fluidic circuits (IFC), a mix was prepared consisting of 440 μL 2X TaqMan Master Mix (Applied Biosystem, 4,369,016), 44 μL 20× DNA Binding Dye Sample Loading Reagent (100–7609, Fluidigm), 44 μl 20X Evagreen (31,000, Biotium) plus 132 μL TE, and 6 μL of this mix was dispensed to each well of a 96-well assay plate. Two microliter of preamplified and diluted cDNA sample was added to each well and the plate was briefly vortexed and centrifuged. For the assays, 5 μL of each Assay (5 μM each primer in primer-mix (2X assay loading reagent (100–7611, Fluidigm) and Tris EDTA) were dispensed to each Detector Inlet of the 96.96 IFC. Following priming of the IFC in the IFC Controller HX, 5 μL of the cDNA sample + reagent mix and 5 μl of Assay were dispensed to each Sample Inlet of the 96.96 IFC. After loading the assays and samples into the IFC in the IFC Controller HX, the IFC was transferred to the BioMark and PCR was performed using the following thermal protocol: Thermal Mix of 50 °C, 2 min; 70 °C, 30 min; 25 °C, 10 min, Hot Start at 50 °C, 2 min; 95 °C, 10 min, PCR Cycle of 35 cycles of (95 °C, 15 s; 60 °C, 60 s), and Melting analysis (60 °C, 30s; 95 °C,1 °C/3 s). Results were analyzed using the Fluidigm real-time PCR analysis software v.4.1.3.

### Data pre-processing

The first part of the analysis is to clean up the data with the Fluidigm real-time PCR analysis software v.4.1.3. Data were pre-processed for expression analysis as follows: the cycle threshold (Ct) values registered from amplifications that generated melting curves with aberrant Tm (melting temperature) or with products giving rise to a double peak in melting curves (corresponding to a mixture of expected and aberrant PCR products) were removed.

### Gene expression analysis

The selectHKgenes function with the “Vandesompele” method of the SLqPCR package was used with RStudio (Version 1.2.1335) to choose the five most stable housekeeping genes. The five housekeeping genes for the relative quantification of mRNA levels of target genes were SDHA, GLUT8, PDHA1, POL2 and Luciferase. Luciferase is an exogenous RNA (Promega), added to each sample during the reverse transcription (100 pg per inch) to allow normalization of the data, as previously described [50, 51]. The slope of a standard curve using serial dilutions of cDNA measured the efficiency (E) of PCR. In all cases, PCR efficiency values ranged between 1.90 and 2. The analyses were done with RStudio [52, 53] with:

$$ \mathrm{Relative}\kern0.5em \mathrm{gene}\kern0.5em \mathrm{expression}=\frac{{\left({E}_{\mathrm{target}}\right)}^{\Delta {\mathrm{Ct}}_{\mathrm{target}}}}{\mathrm{geomean}\kern0.5em \Big({\left({E}_{\mathrm{ref}}\right)}^{\Delta {\mathrm{Ct}}_{\mathrm{ref}}}} $$

$$ \mathrm{Relative}\kern0.5em \mathrm{gene}\kern0.5em \mathrm{expression}=\frac{2^{\Delta {\mathrm{Ct}}_{\mathrm{target}}}}{{\left({2}^{\Delta {\mathrm{Ct}}_{\mathrm{SDHA}}}\times {2}^{\Delta {\mathrm{Ct}}_{\mathrm{GLUT}8}}\times {2}^{\Delta {\mathrm{Ct}}_{\mathrm{PDHA}1}}\times {2}^{\Delta {\mathrm{Ct}}_{\mathrm{POL}2}}\times {2}^{\Delta {\mathrm{Ct}}_{\mathrm{luciferase}}}\right)}^{\frac{1}{5}}} $$

$$ \mathrm{Ct}=\mathrm{threshold}\ \mathrm{cycle} $$

$$ {\Delta \mathrm{Ct}}_{\mathrm{target}}={\mathrm{Ct}}_{\mathrm{control}}-{\mathrm{Ct}}_{\mathrm{sample}} $$

$$ {\Delta \mathrm{Ct}}_{\mathrm{ref}}={\mathrm{Ct}}_{\mathrm{control}}-{\mathrm{Ct}}_{\mathrm{sample}} $$

$$ {\mathrm{Ct}}_{\mathrm{control}}=\mathrm{average}\ \mathrm{Ct}\ \mathrm{of}\ \mathrm{all}\ \mathrm{samples} $$

### Statistical analysis

Statistical analyses were done using the Graphpad Prism version 8 for Windows (GraphPad software, La Jolla California USA, www.graphaapd.com (serial number *GP8–1598457-RJQD-5E2EC*)). Data are presented with a box-and-whisker plot, boxes ranging from the 25th to the 75th percentiles, and whiskers ranging from the lowest to the highest value. When the data set presented a Normal distribution (assessed by Shapiro–Wilk test), parametric variance analysis (ANOVA) was performed followed by a Bonferroni’s multiple comparison test as post hoc analysis. When normal distribution was not demonstrated, the Kruskal–Wallis non-parametric test was performed followed by a Dunn’s test as post hoc analysis. In every case, differences between the groups were considered statistically significant if the value of *P* < 0.05.

The heatmap.2 function from the gplots package was used to draw heatmaps with RStudio. The **corrplot** package was used to draw the correlation matrix; this package contains algorithms to reorder the matrix according to the degree of correlation between the variables.

## Supplementary information

**Additional file 1: Supplemental Table 1.**. Statistical summary of developmental gene expression comparisons over time from E12 to D4. Statistical comparisons over time (E12 to D4) of the gene expressions illustrated in Fig. 1. Depending on shapiro test result, ANOVA with Bonferroni’s multiple comparisons test or Kruskal-Wallis (K-W) with Dunn’s multiple comparisons test were used (*n* = 14–20). ns: not significant, *: *P* < 0.05, **: *P* < 0.01, ***: *P* < 0.001, ****: *P* < 0.0001. **Supplemental Table 2.** Statistical summary of carbohydrate-related gene expression comparisons over time from E12 to D4. Statistical comparisons over time (E12 to D4) of the gene expressions illustrated in Fig. 2. Depending on shapiro test result, ANOVA with Bonferroni’s multiple comparisons test or Kruskal-Wallis (K-W) with Dunn’s multiple comparisons test were used (*n* = 7–20). ns: not significant, *: *P* < 0.05, **: *P* < 0.01, ***: *P* < 0.001, ****: *P* < 0.0001. **Supplemental Table 3.** Statistical summary of lipid-related gene expression comparisons over time from E12 to D4. Statistical comparisons over time (E12 to D4) of the gene expressions illustrated in Fig. 3. Depending on shapiro test result, ANOVA with Bonferroni’s multiple comparisons test or Kruskal-Wallis (K-W) with Dunn’s multiple comparisons test were used (*n* = 10–20). ns: not significant, *: *P* < 0.05, **: *P* < 0.01, ***: *P* < 0.001, ****: *P* < 0.0001. **Supplemental Table 4.** Statistical summary of stress-related gene expression comparisons over time from E12 to D4. Statistical comparisons over time (E12 to D4) of the gene expressions illustrated in Fig. 4. Depending on shapiro test result, ANOVA with Bonferroni’s multiple comparisons test or Kruskal-Wallis (K-W) with Dunn’s multiple comparisons test were used (*n* = 16–20). ns: not significant, *: *P* < 0.05, **: *P* < 0.01, ***: *P* < 0.001, ****: *P* < 0.0001. **Supplemental Table 5.** Informative table on primers used for the study of development-related genes. **Supplemental Table 6.** Informative table on primers used for the study of carbohydrate-related genes. **Supplemental Table 7.** Informative table on primers used for the study of lipid-related genes. **Supplemental Table 8.** Informative table on primers used for the study of stress-related genes.

## Data Availability

The datasets analyzed during the current study are available in the NCBI Gene Expression Omnibus (GEO) repository, with accession number GSE157687 (Real-time PCR analysis during mule duck development). These data also include the accession numbers of all the genes studied in this project.

## Abbreviations

ANOVA: Analysis of variance
Ct: Cycle threshold
D4: Day 4 after hatching
E12: Embryonic day 12
PCR: Polymerase chain reaction
Tm: Melting temperature
