# Supplementary material for: Ontogeny of hepatic metabolism in mule ducks highlights different gene expression profiles between carbohydrate and lipid metabolic pathways
Source: BMC Genomics. 2020 Oct 27;21:742. doi: 10.1186/s12864-020-07093-w (PMC7590481; doi:10.1186/s12864-020-07093-w)
Supplement: Supplementary file 1 — Additional file 1: Supplemental Table 1.. Statistical summary of developmental gene expression comparisons over time from E12 to D4. Statistical comparisons over time (E12 to D4) of the gene expressions illustrated in Fig. 1. Depending on shapiro test result, ANOVA with Bonferroni’s multiple comparisons test or Kruskal-Wallis (K-W) with Dunn’s multiple comparisons test were used (n = 14–20). ns: not significant, *: P < 0.05, **: P < 0.01, ***: P < 0.001, ****: P < 0.0001. Supplemental Table 2. Statistical summary of carbohydrate-related gene expression comparisons over time from E12 to D4. Statistical comparisons over time (E12 to D4) of the gene expressions illustrated in Fig. 2. Depending on shapiro test result, ANOVA with Bonferroni’s multiple comparisons test or Kruskal-Wallis (K-W) with Dunn’s multiple comparisons test were used (n = 7–20). ns: not significant, *: P < 0.05, **: P < 0.01, ***: P < 0.001, ****: P < 0.0001. Supplemental Table 3. Statistical summary of lipid-related gene expression comparisons over time from E12 to D4. Statistical comparisons over time (E12 to D4) of the gene expressions illustrated in Fig. 3. Depending on shapiro test result, ANOVA with Bonferroni’s multiple comparisons test or Kruskal-Wallis (K-W) with Dunn’s multiple comparisons test were used (n = 10–20). ns: not significant, *: P < 0.05, **: P < 0.01, ***: P < 0.001, ****: P < 0.0001. Supplemental Table 4. Statistical summary of stress-related gene expression comparisons over time from E12 to D4. Statistical comparisons over time (E12 to D4) of the gene expressions illustrated in Fig. 4. Depending on shapiro test result, ANOVA with Bonferroni’s multiple comparisons test or Kruskal-Wallis (K-W) with Dunn’s multiple comparisons test were used (n = 16–20). ns: not significant, *: P < 0.05, **: P < 0.01, ***: P < 0.001, ****: P < 0.0001. Supplemental Table 5. Informative table on primers used for the study of development-related genes. Supplemental Table 6. Informative table on pr [file 12864_2020_7093_MOESM1_ESM.docx]

**Supplemental tables**

**Supplemental Table 1. Statistical summary of developmental gene expression comparisons over time from E12 to D4**

Statistical comparisons over time (E12 to D4) of the gene expressions illustrated in figure 1.

Depending on shapiro test result, ANOVA with Bonferroni’s multiple comparisons test or Kruskal-Wallis (K-W) with Dunn’s multiple comparisons test were used (n=14-20).

ns: not significant, *: *P* < 0.05, **: *P* < 0.01, ***: *P* < 0.001, ****: P<0.0001.

**Supplemental Table 2. Statistical summary of carbohydrate-related gene expression comparisons over time from E12 to D4**

Statistical comparisons over time (E12 to D4) of the gene expressions illustrated in figure 2.

Depending on shapiro test result, ANOVA with Bonferroni’s multiple comparisons test or Kruskal-Wallis (K-W) with Dunn’s multiple comparisons test were used (n=7-20).

ns: not significant, *: *P* < 0.05, **: *P* < 0.01, ***: *P* < 0.001, ****: P<0.0001.

**Supplemental Table 3. Statistical summary of lipid-related gene expression comparisons over time from E12 to D4**

Statistical comparisons over time (E12 to D4) of the gene expressions illustrated in figure 3.

Depending on shapiro test result, ANOVA with Bonferroni’s multiple comparisons test or Kruskal-Wallis (K-W) with Dunn’s multiple comparisons test were used (n=10-20).

ns: not significant, *: *P* < 0.05, **: *P* < 0.01, ***: *P* < 0.001, ****: P<0.0001.

**Supplemental Table 4. Statistical summary of stress-related gene expression comparisons over time from E12 to D4**

Statistical comparisons over time (E12 to D4) of the gene expressions illustrated in figure 4.

Depending on shapiro test result, ANOVA with Bonferroni’s multiple comparisons test or Kruskal-Wallis (K-W) with Dunn’s multiple comparisons test were used (n=16-20).

ns: not significant, *: *P* < 0.05, **: *P* < 0.01, ***: *P* < 0.001, ****: P<0.0001.

**Supplemental Table 5**

Informative table on primers used for the study of development-related genes.

**Supplemental Table 6**

Informative table on primers used for the study of carbohydrate-related genes.

**Supplemental Table 7**

Informative table on primers used for the study of lipid-related genes.

**Supplemental Table 8**

Informative table on primers used for the study of stress-related genes.
